# Treatment of Epistaxis by a Sponge Plug

**Published:** 1899-11-18

**Authors:** 


### TREATMENT OF EPISTAXIS BY A SPONGE PLUG.

Dr. Cornick, writing in the *New York Medical Journal*, suggests the treatment of epistaxis by the insertion of a piece of sponge into the affected nostril. It is essential that the sponge shall be thoroughly squeezed dry so that some expansion shall take place. All that he has found necessary to control the bleeding has been to cut with a pair of scissors a dry plug of sponge comparable in size and length to the little finger of a twelve-year-old boy. This should be thoroughly soaked in boiled water, squeezed dry, and inserted gently for its full length along the floor of the bleeding nostril. The expansive pressure of the sponge, aided by the coagulation of the blood in its interstices, will, he says, check the bleeding at once. It should be removed in twelve hours, and under no circumstances should it be allowed to remain more than twenty-four hours. This would seem to be a modification of the discarded treatment by the sponge tent, the tent in this case being already softened and considerably expanded by moisture before being inserted. Our readers need hardly be reminded that the same object, of bringing a somewhat expansible plug to bear upon the bleeding point, has in other hands been accomplished by aid of an india-rubber bag which could be expanded by bellows to the required degree. This plan possesses the great advantage that the plug can be easily removed, but it may be doubted whether the india-rubber will take the exact form of the nostril with such accuracy as the sponge plug now suggested.

